# Unintentional miRNA Ablation Is a Risk Factor in Gene Knockout Studies: A Short Report

**DOI:** 10.1371/journal.pgen.0040034

**Published:** 2008-02-15

**Authors:** Ivan Osokine, Ruby Hsu, Gabriel B Loeb, Michael T McManus

**Affiliations:** Department of Microbiology and Immunology, University of California San Francisco (UCSF) Diabetes Center, University of California San Francisco, San Francisco, California, United States of America; Stanford University School of Medicine, United States of America

## Abstract

One of the most powerful techniques for studying the function of a gene is to disrupt the expression of that gene using genetic engineering strategies such as targeted recombination or viral integration of gene trap cassettes. The tremendous utility of these tools was recognized this year with the awarding of the Nobel Prize in Physiology or Medicine to Capecchi, Evans, and Smithies for their pioneering work in targeted recombination mutagenesis in mammals. Another noteworthy discovery made nearly a decade ago was the identification of a novel class of non-coding genes called microRNAs. MicroRNAs are among the largest known classes of regulatory elements with more than 1000 predicted to exist in the mouse genome. Over 50% of known microRNAs are located within introns of coding genes. Given that currently about half of the genes in mouse have been knocked out, we investigated the possibility that intronic microRNAs may have been coincidentally deleted or disrupted in some of these mouse models. We searched published murine knockout studies and gene trap embryonic stem cell line databases for cases where a microRNA was located within or near the manipulated genomic loci, finding almost 200 cases where microRNA expression may have been disrupted along with another gene. Our results draw attention to the need for careful planning in future knockout studies to minimize the unintentional disruption of microRNAs. These data also raise the possibility that many knockout studies may need to be reexamined to determine if loss of a microRNA contributes to the phenotypic consequences attributed to loss of a protein-encoding gene.

## Author Summary

To determine the function of a gene, it is often informative to first disrupt the expression of that gene through targeted recombination or the insertion of gene trap cassettes. In our study, we point out that these approaches may be confounded by the presence of small non-coding elements known as microRNAs. MicroRNAs constitute one of the largest classes of regulatory elements, and over 50% of known microRNAs have been identified within an intron of a coding gene. Disruption of a gene could therefore also result in the disruption of microRNAs in the region. In this study, we searched databases of gene-trapped cell lines as well as previously published knockout studies and report almost 200 examples where microRNA expression may have been unintentionally disrupted. Our results are of broad interest and importance because they raise the possibility that a number of protein function studies may need to be reexamined to determine whether the loss of a microRNA may have contributed to the phenotype previously attributed to the loss of a protein.

## Introduction

In the mouse, stable disruption of a gene is typically accomplished using gene trap mutagenesis or targeted homologous recombination. We wish to communicate the overlooked possibility of unintentionally disrupting microRNA (miRNA) genes along with a targeted gene. Because miRNAs play key roles in many cellular processes, the unintended ablation of these species may have significant consequences that complicate the interpretation of gene knockout studies.

## Methods

**Figure 1:**
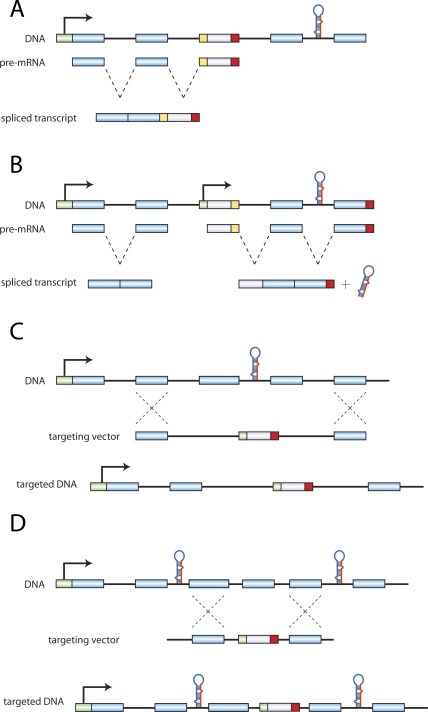
Configurations of Protein:miRNA Gene Disruptions. Retroviral gene traps have the potential to (A) ablate miRNA transcription or (B) lead to constitutive overexpression of the miRNA. (C) Schematic of a recombinant gene-targeting strategy whereby an intronic miRNA is ablated or (D) the miRNA is upstream and/or downstream of the targeting cassette. Promoter, green; exon, blue; selection marker, white; splice acceptor or donor, yellow; transcriptional terminator, red.

Given that many miRNAs are located within introns of longer coding transcripts, we reasoned that a gene trap disrupting a host gene could also alter miRNA expression in one of two ways. The trapping cassette could either ablate miRNA expression with a terminal polyadenylation sequence (Figure 1A) or overexpress an miRNA via an internal promoter (Figure 1B). To determine the potential extent of these unintended changes in miRNA expression, we compared the genomic position all mouse gene traps listed in the International Gene Trap Consortium (IGTC) [1] to the loci of 367 annotated mouse miRNA genes as well as candidate miRNA genes computationally identified by Berezikov et al., 28% of which have been validated to date [2,3]. In the cases where an miRNA was located within an intron of a host gene, we identified any gene traps which inserted within the host gene transcript and upstream of the miRNA. Using the same set of annotated and candidate miRNAs, we next identified all protein-coding genes with an miRNA located within the transcribed loci, in either the sense or the antisense orientation. We cross-referenced these genes with all homologous recombination studies listed in the Mouse Genome Informatics (MGI) database (v. 3.54) [4] to assemble a list of studies where the miRNA and coding gene were potentially co-ablated (Table S1). The boundaries of the deleted loci were bioinformatically verified for each study.

## Results/Discussion

Our analysis of the IGTC database revealed 98 annotated or candidate miRNAs potentially misregulated in 420 gene trap cell lines (Table S2). A study of the *slit3* gene [5] is an example of a potential unintended double-knockout scenario produced from a gene trap cell line. To ablate *slit3*, the authors used a trap located upstream of exon 6, which produced a truncated slit3 mRNA. Mir-218–2 is located within intron 14 of *slit3*, and the potential loss of mir-218–2 expression may contribute to the phenotype resulting from the loss of functional slit3.

The analysis of the MGI database yielded a small but significant number of studies where miRNAs may have been unintentionally disrupted (Table S1). In addition to 20 studies where an annotated or candidate miRNA was completely ablated by the targeting strategy (Figure 1C), there were also numerous studies describing the deletion of regions immediately upstream (78 cases) or downstream (55 cases) of a miRNA (Figure 1D), or in the promoter of the host gene (4 cases). MiRNAs have been shown to be transcribed in conjunction with a host transcript or from an independent promoter [6]. Therefore, the disruption of host promoters or of regions adjacent to miRNAs may compromise promoter and/or enhancer sites for these miRNAs.

While 71 of the studies in our analysis were published prior to the expansion of the miRNA field in 2002, the fact that 90 were published since may indicate that miRNAs in targeted loci continue to be overlooked. To avoid inadvertent double-knockout scenarios, we wish to alert investigators to consider non-coding elements in the locus to be deleted. Because not all non-coding elements have been annotated, it may be preferable to employ methods that minimize the deletion of endogenous DNA. We also wish to raise the interesting possibility that a number of studies may need to be reevaluated to dissociate the consequences of ablating an miRNA from the consequences of ablating the targeted gene.

## Supporting Information

Table S1 Recombination Locus on Coding Gene Strand with respect to miRNA (40 KB XLS)

Table S2 Gene Traps Located Within or Immediately Upstream of Genes Containing Intragenic miRNAs (64 KB XLS)

## References

[pgen-0040034-b001] Griffiths-Jones S, Grocock RJ, van Dongen S, Bateman A, Enright AJ (2006). miRBase: microRNA sequences, targets and gene nomenclature. Nucleic Acids Res.

[pgen-0040034-b002] Berezikov E, Goryev V, van de Belt J, Wienholds E, Plasterk RH (2005). Phylogenetic shadowing and computational identification of human microRNA genes. Cell.

[pgen-0040034-b003] Nord AS, Chang PJ, Conklin BR, Cox AV, Harper CA (2006). The International Gene Trap Consortium Website: a portal to all publicly available gene trap cell lines in mouse. Nucleic Acids Res.

[pgen-0040034-b004] Eppig JT, Bult CJ, Kadin JA, Richardson JE, Blake JA, and the members of the Mouse Genome Database Group (2005). The Mouse Genome Database (MGD): from genes to mice—a community resource for mouse biology. Nucleic Acids Res.

[pgen-0040034-b005] Liu J, Zhang L, Wang D, Shen H, Jiang M (2003). Congenital diaphragmatic hernia, kidney agenesis and cardiac defects associated with Slit3-deficiency in mice. Mech Dev.

[pgen-0040034-b006] Rodriguez A, Griffiths-Jones S, Ashurst JL, Bradley A (2004). Identification of mammalian microRNA host genes and transcription units. Genome Res.

